# Testing Multiple Methods to Effectively Promote Use of a Knowledge Portal to Health Policy Makers: Quasi-Experimental Evaluation

**DOI:** 10.2196/41997

**Published:** 2023-06-28

**Authors:** Matthew Weber, Veronica L Armour, Calandra Lindstadt, Itzhak Yanovitzky

**Affiliations:** 1 Department of Communication School of Communication and Information Rutgers University New Brunswick, NJ United States; 2 Office of Engagement and Extension Colorado State University Grand Junction, CO United States

**Keywords:** depression, depression screening, policy making, Google Ads, analytics, knowledge brokers, knowledge sharing, online, resources, teen, young adult, effectiveness

## Abstract

**Background:**

Health policy makers and advocates increasingly utilize online resources for policy-relevant knowledge. Knowledge brokering is one potential mechanism to encourage the use of research evidence in policy making, but the mechanisms of knowledge brokerage in online spaces are understudied. This work looks at knowledge brokerage through the launch of Project ASPEN, an online knowledge portal developed in response to a New Jersey legislative act that established a pilot program for adolescent depression screening for young adults in grades 7-12.

**Objective:**

This study compares the ability to drive policy brief downloads by policy makers and advocates from the Project ASPEN knowledge portal using a variety of online methods to promote the knowledge portal.

**Methods:**

The knowledge portal was launched on February 1, 2022, and a Google Ad campaign was run between February 27, 2022, and March 26, 2022. Subsequently, a targeted social media campaign, an email campaign, and tailored research presentations were used to promote the website. Promotional activities ended on May 31, 2022. Website analytics were used to track a variety of actions including new users coming to the website, page views, and policy brief downloads. Statistical analysis was used to assess the efficacy of different approaches.

**Results:**

The campaign generated 2837 unique user visits to the knowledge portal and 4713 page views. In addition, the campaign generated 6.5 policy web page views/day and 0.7 policy brief downloads/day compared with 1.8 views/day and 0.5 downloads/day in the month following the campaign. The rate of policy brief page view conversions was significantly higher for Google Ads compared with other channels such as email (16.0 vs 5.4; *P*<.001) and tailored research presentations (16.0 vs 0.8; *P*<.001). The download conversion rate for Google Ads was significantly higher compared with social media (1.2 vs 0.1; *P*<.001) and knowledge brokering activities (1.2 vs 0.2; *P*<.001). By contrast, the download conversion rate for the email campaign was significantly higher than that for social media (1.0 vs 0.1; *P*<.001) and tailored research presentations (1.0 vs 0.2; *P*<.001). While Google Ads for this campaign cost an average of US $2.09 per click, the cost per conversion was US $11 per conversion to drive targeted policy web page views and US $147 per conversion to drive policy brief downloads. While other approaches drove less traffic, those approaches were more targeted and cost-effective.

**Conclusions:**

Four tactics were tested to drive user engagement with policy briefs on the Project ASPEN knowledge portal. Google Ads was shown to be effective in driving a high volume of policy web page views but was ineffective in terms of relative costs. More targeted approaches such as email campaigns and tailored research presentations given to policy makers and advocates to promote the use of research evidence on the knowledge portal website are likely to be more effective when balancing goals and cost-effectiveness.

## Introduction

### Background

In late 2021, New Jersey Governor Phil Murphy signed NJ A970 into law, launching the Mental Health Screening in Schools Grant Program, a US $1 million grant program designed to allow districts to implement depression screening programs to identify students in grades 7 through 12 who are at risk for depression. This passage was followed by a period of implementation beginning in 2021. Project ASPEN (Active Surveillance of Policy Ecosystems and Networks) was a research project funded to assess state policy makers’ and mental health policy advocates’ use of research evidence in depression-related policy making and implementation. Based on consultation with parent advocates and nonprofit organizations, and an extensive review of available research, it was clear that there is a lack of original research that explores potential barriers to implementation from the perspective of multiple stakeholders, including parents and school personnel.

The Project ASPEN knowledge portal was launched as a website to broker and connect potential users with policy implementation–relevant research regarding school-based adolescent depression screening. A quasi-experimental design was implemented to compare the relative utility of different research dissemination strategies based on the evidence included in the knowledge portal.

### Knowledge Brokerage

Knowledge brokers are individuals or organizations that act as intermediaries facilitating the spread of knowledge through formal and informal networks [1,2]. Prior scholarship establishes knowledge brokers as potentially effective actors for translating research into practice to inform evidence-based decision-making, with varying evidence of effectiveness across practice and policy contexts [3,4]. The concept of knowledge brokerage is particularly salient in clinical settings [5], where knowledge brokers are seen as enabling the translation of research evidence into practice. Further, there are different types of knowledge brokering activities, including driving awareness, improving accessibility, facilitating engagement, linking knowledge to relevant actors, and mobilizing knowledge to drive action [6].

Knowledge brokers can be effective agents of knowledge transfer because they are able to leverage their existing relationships with policy makers to improve the likelihood that research evidence, as well as other inputs, is considered in policy-making processes. The actual knowledge transfer functions that knowledge brokers perform depend on their capacity and opportunities to acquire, interpret, and disseminate research evidence. This may be easier to accomplish in practice settings (ie, facilitating the adoption of evidence-based practices in clinical settings) [7], but is more challenging in policy settings given the large number of decision makers and stakeholders involved, the fact that research is only 1 form of input considered in policy, and the complexity of the policy-making process [8]. Therefore, knowledge brokers who are already active in the policy space may be in a better position to broker policy-relevant research evidence to policy decision makers than those who try to influence policy making from the outside.

Digital knowledge platforms can support knowledge brokerage in the policy domain by providing timely, reliable, and unrestricted access to policy-relevant research evidence to policy makers, intermediaries and stakeholders, journalists, and other potential users of research [9]. In practice settings, knowledge brokerage may be able to perform several key knowledge functions including needs assessments, knowledge management, knowledge translation and exchange, network development and maintenance, and facilitation of evidence-informed decision-making processes [10]. Knowledge brokers in the policy domain rarely have the capacity or the resources to perform similar functions, and knowledge portals can make it easier for them to find and use relevant research evidence that is already translated and packaged for consumption [11]. Early attempts to utilize online tools for knowledge transfer focused on reinforcing existing knowledge sharing routines [12]. Recent work in dissemination and implementation views knowledge portals and clearinghouses as an integral component of the broader information ecosystems that policy makers and other actors can access for obtaining policy-relevant research evidence [13,14].

This study assessed the use of a newly created knowledge portal intended to support knowledge brokerage activities as a mechanism for improving access to policy-relevant research evidence and other resources. A knowledge portal is broadly defined as an intentionally designed resource that organizes information to provide others with access; most knowledge portals are websites, although a knowledge portal could also be a document repository on a site such as Box (Box, Inc.) or Dropbox (Dropbox, Inc.). The common components of knowledge portals are content management systems, knowledge repositories, search engine, applications and services, collaboration and communication tools, personalization and user accounts, and an integrated and aesthetically pleasing interface [15]. However, knowledge portals are only useful if potential users are aware they exist. Thus, whereas considerable investments of thought, time, and resources go into the design and deployment of knowledge portals, it is equally important to consider how potential users learn about and are encouraged to interact with a knowledge portal.

This study assessed the use of a newly created knowledge portal that curates research evidence and other resources regarding the topic of screening adolescents for depression and suicide by tracking users’ engagement. The study, in addition, used a pretest-posttest quasi-experimental design to compare the relative utility of different strategies for driving web traffic to the portal in general and to specific content (policy briefs) that was hypothesized to be of most interest to legislators and advocates involved in policy making regarding the implementation of school-based depression screening.

### Knowledge Portals for Brokerage

Policy makers and policy advocates often struggle to identify relevant policy information, or identify ineffective evidence [15]. An extensive meta-analysis of research on effective strategies for dissemination of research underscored the importance of brokering activities, and the development of resources to help policy makers navigate complex information environments [16]. Individuals and organizations can serve a key role as brokers in health policy settings [1]. Knowledge brokers leverage website platforms to support organizational and advocacy-oriented goals as well as to engage in direct conversation with audiences [17,18]. Recent work uses the term platformed knowledge brokerage to capture the process whereby online entities act as a “surrogate,” and support the knowledge brokerage work performed by human actors or organizations [9]. Websites can support knowledge brokerage by serving as a critical resource, functioning as an intermediary that links producers of knowledge with the use of knowledge needed for implementation [19].

### Driving Engagement With Websites as Brokers

To drive engagement with policy makers and other key stakeholders such as policy advocates, knowledge portals must intentionally synthesize and translate relevant knowledge. Extant research demonstrates that knowledge portals such as Patient-centric Research Engagement Portals can be effective for communicating research to public audiences when it is clear there is a need on the part of the audience, and intentional design to help communicate critical information [20]. Thus, there must be an intentional alignment with audience needs, the research and information presented must be robust, and policy makers should be presented with a spectrum of information and relevant policy actions [21]. Further, there needs to be trust between consumers of research and producers of research, and there should be intentionality when leveraging multiple forms of technology [22]. The use of websites as knowledge brokers offers benefits such as capturing implicit knowledge and dissemination of information [23].

A central component of knowledge portals is intentionally designing to support brokerage of research evidence. The most common components of knowledge portals are content management databases, knowledge repositories, search functions, embedded applications and services, collaboration and communication tools, personalization and user accounts, and an integrated and aesthetically pleasing interface [24,25], which lend credence to the notion that websites can support knowledge sharing as part of the knowledge brokering process.

### Metrics and Measurement

Generally, website use is measured based on visits, pages per visit, length of visit, bounce rate, and return rate [26]. These metrics are used to gauge access to a website or to information but cannot directly assess use. Additional metrics utilized are the number of pages a user views per visit, the average amount of time a user spends on a website per visit, the bounce rate (percentage of users who leave a website after viewing only 1 page), and click-through rate (percentage of users clicking on a hyperlink or other component), among other measures [27].

While these types of metrics do not indicate what users will do with the information once it has been accessed, they do provide a measure of the general utility of a website, including the number of users present and the degree to which users navigate across the breadth of a given website. Although ultimately many websites have sought to increase knowledge or to impact health outcomes, this type of activity has proven difficult to measure with existing metrics [28]. In part, website metrics measure user activity on the website, but it is difficult to connect that activity to offline activity (such as the use of knowledge for informing decisions [29]).

### Social Media and Search Utilization

Digital awareness campaigns that make use of social media sites’ pay-per-click advertising are influential in generating site visits [30], although it is unclear what the value of such campaigns is in terms of driving action. Despite the increase in site visits that advertisement campaigns facilitate, the quality of visits is often lower than visits generated by other approaches, and the user engagement is shallower with less time spent on the site and fewer return visits [31].

Social media is increasingly important for research producers and knowledge brokers as a means of knowledge sharing and for the facilitation of evidence-informed practice [32]. Social media is a channel that increases the reach of a website, or of information more broadly, and enables passive consumption of information by a broader network of individuals [33]. Engagement via social media can facilitate knowledge sharing across organizational boundaries. A pay-per-click approach, executed on social media or via web search sites such as Google, can be effective in driving traffic to a health website [30]. Several studies have demonstrated the potential efficacy of health care campaigns on Google as a means of driving engagement [31].

## Methods

### Evaluations

The study assessed the use of a web-based knowledge portal by a general as well as a specifically targeted group of users. A quasi-experimental evaluation is used to study the use of websites as knowledge portals. Quasi-experimental evaluations are utilized in medical research to study contexts where it is not possible for the researchers to control for the circumstances of the study. Such an evaluation was appropriate here given the public nature of the website and the subsequent inability to assign users to treatment. Policy implementation contexts represent one such circumstance, as it is difficult to control for the circumstances leading to the introduction of a policy solution, and it is similarly difficult to control for the implementation of the policy itself. For example, recent research in this domain used quasi-experimental evaluations to study the implementation of a web-based information system to support family caregivers [34].

### Ethics Considerations

Rutgers Institutional Review Board (IRB) approval was obtained. The plan for data collection and analysis was approved as protocol #2021001939 and the broader project protocols were approved as #2019000782. Summary data are presented to protect individual identities, and the researchers did not retain any individual identifying information.

### Rationale and Development of a Website

The focus of this study was on the design and implementation of a knowledge portal to support knowledge brokering regarding the implementation of school-based adolescent depression screening policy in New Jersey in 2021 and 2022.

Rates of depression increase notably during adolescence, and there is a strong association between adolescent depression and adverse outcomes including suicide, educational underachievement, and psychopathology [35]. Screening for depression among adolescents is recommended by the US Preventive Services Task Force guidelines for adolescents aged 12-18 [36], but overall rates of screening are low [37]. Screening has proved tough to implement consistently in primary care settings, and a shortage of mental health professionals nationally acerbates the problem [38]. School-based screening can potentially close existing gaps in adolescent depression screening, particularly for adolescents from underserved social groups [39].

In late 2021, New Jersey passed NJ A970 (S2259), launching a pilot program to assess the feasibility of implementing school-based adolescent depression screening program according to current screening guidelines. The legislation empowers the New Jersey Department of Education and the Department of Human Services to develop a standard screening protocol, staffing requirements, and reporting standards that applicant schools must implement to receive funding. In consultation with local youth mental health advocates, school administrators, school psychologists and social workers, and parents of adolescents in public schools, the research team conducted a series of studies to identify major barriers and facilitators to implementation, and findings were shared in a form of policy briefs made available on the knowledge portal created to promote use of research evidence for the purpose of informing planning for implementation.

### Portal Design

The knowledge portal was designed as a component of Project ASPEN in collaboration with the National Alliance on Mental Illness, New Jersey, a major broker of research on youth mental health to state government. The collaboration focused on identifying research evidence that is most relevant to implementation (eg, choice of a screening instrument, resources and training needed to support implementation in schools, parental concerns regarding screening) that can be included, additional useful online resources that can be curated on the website, and user-centered design considerations that will make the portal useful for diverse audiences.

In organizing the resources for the online knowledge portal, different stages of user interaction with the site were considered, from preliminary research on adolescent depression screening to seeking information that is relevant to policy implementation. Given that the policy-making process progressed to the policy implementation planning phase, the primary users were identified as implementation stakeholders (advocates, school boards, and professional associations), including state government officials. Additional users include policy makers, specifically the legislative staffers, state assembly members, and state senators who are involved in the overarching legislative process.

Knowledge brokering activities enabled by the website needed to focus on the implementation of the proposed depression screening protocol. With the user needs identified within the implementation stage of the legislation, we focused on organizing the categories of resources to emphasize and aid information-seeking needs related to the different aspects of screening implementation. Resources were organized into categories including treatments, toolkits, guidelines, and assessment. An information librarian joined the project team to conduct an extensive review of available national and state resources relevant to the issue of adolescent depression screening. In addition to resource collection, the research team produced original research findings that were summarized in 4 research briefs that address core implementation challenges related to statewide adolescent depression screening in schools. The briefs ranged in length from 3 to 7 pages. The titles of those policy briefs are as follows:

- New Jersey Parents’ Views of Adolescent Depression Screening
- Call for Action on Adolescent Depression: What Do Schools In New Jersey Need to Identify and Support Students at Risk for Depression?
- Adolescent Depression Screening: Exploring Barriers and Facilitators of Implementation in School Settings
- Adolescent Depression Screening Instruments: A Review of Existing Instruments to Screen for Adolescent Depression

**Figure 1:**
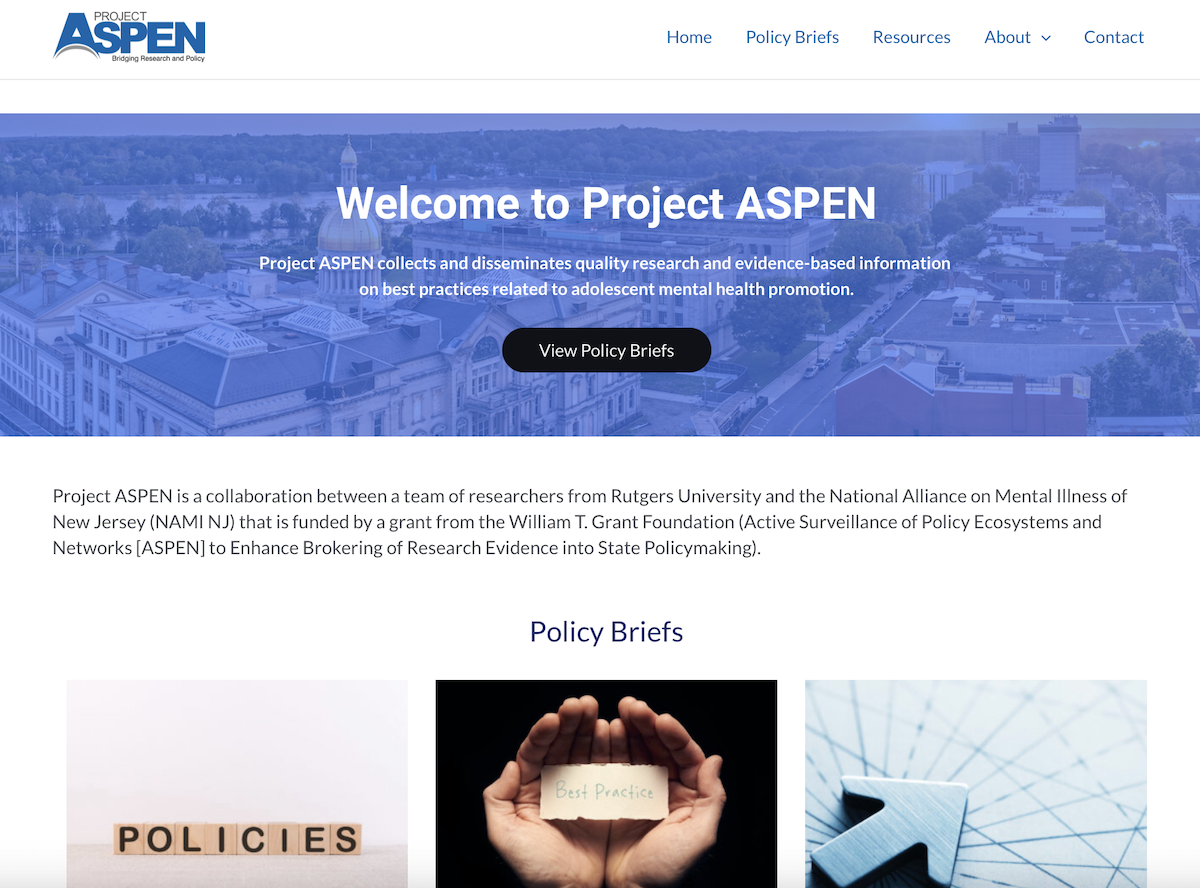
Screenshot of the Project ASPEN website homepage.

A graphic web designer joined the project team to support development of the website. Both resource collection and website design were iterative processes that began in September 2021 and concluded in January 2022. The front page of the Project ASPEN website is shown in Figure 1.

### Dissemination Campaign Design

The Project ASPEN website was launched on February 1, 2022, after an extensive design process that began in September 2021. After the initial website launch, a quasi-experimental evaluation testing the efficacy of the Project ASPEN web portal was launched in March 2022 and concluded at the end of May 2022. The quasi-experimental evaluation using a single-site pre-post design without a control group was conducted focusing on the dissemination of the knowledge portal in stages; each stage represented a distinct phase in the launch, and phases were designed to avoid time overlap with one another to isolate measurement of each phase. Building off prior approaches established from our review of recent literature, 4 different strategies were piloted for promoting access to policy briefs [31], followed by a postlaunch period during which data were collected for comparison.

**Figure 2:**
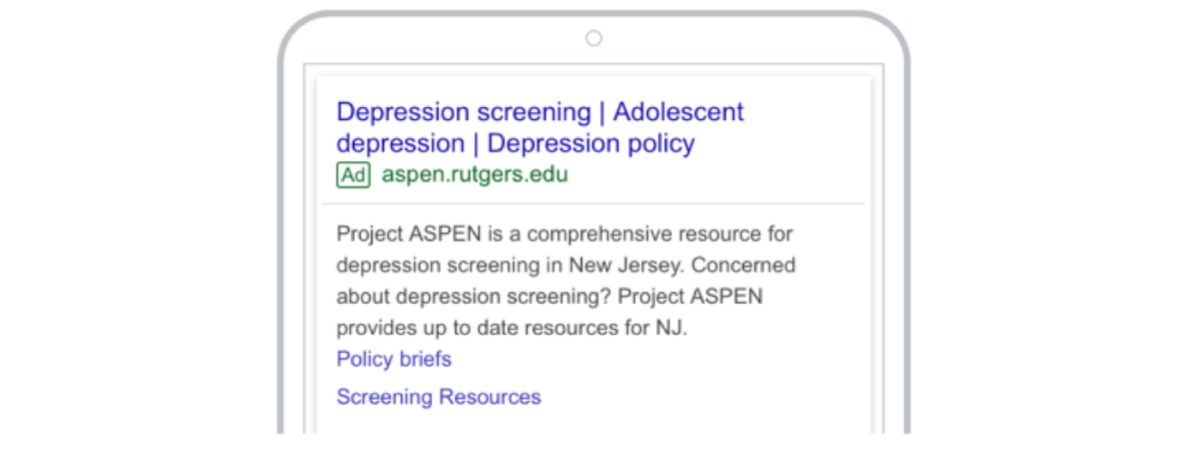
Sample Google Search advertisement.

The first phase was a Google Ads (Google, Inc./Alphabet, Inc.) campaign that ran from February 27, 2022, to March 26, 2022, and focused on driving traffic to the project website via a Google Ads purchase. Keywords are priced based on a system of supply and demand, paired with the specificity of the keywords and other factors such as geographic targeting. A sample of the type of Google Ad used in this promotion is shown in Figure 2.

The campaign used the search network with display functionality from Google Ads; this shows the advertisement when a user searches for selected Google key terms that were selected by the research team. Google Ad functions on a cost-per-click model; advertising was geographically restricted to New Jersey. Keywords were selected based on a review of keyword terms that appear in Google searches paired with a review of key terms on the website portal and in the policy briefs being promoted.

The second phase was targeted dissemination via social media and email. Dissemination via social media ran from April 3 to April 16, 2022, and focused on distribution via Twitter (Twitter, Inc.) and Facebook (Meta Platforms, Inc.). The email campaign was disseminated via the Constant Contact platform and ran from April 24 to April 30. The third and final stage of dissemination was via targeted dissemination activities. The postlaunch comparison period ran from June 1 to July 2. Project leaders gave presentations to key partner organizations and tracked activity on the project website that resulted from these intentional dissemination activities; engagement was tracked by measurement website activity via a customized URL.

**Figure 3:**
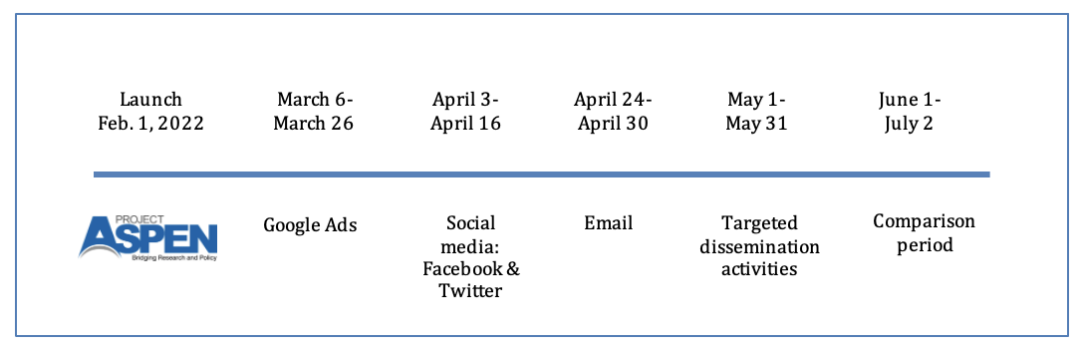
Timeline of key activities for Project ASPEN launch.

Following the aforementioned planned activities, the website was tracked for an additional month to provide a comparison period during which no intentional promotional activity took place. The timeline of the project launch and the associated phases of the quasi-experimental evaluation are illustrated in Figure 3.

Visits to the Project ASPEN website were compared across the different periods of marketing activity. Further, Google Analytics (Google, Inc./Alphabet, Inc.) was used to track user behavior on the website, with the key outcome variable being downloads of research briefs from the website. Key terms related to the promotional activity, Google Ad, and key measures are defined in Table 1.

**Table 1:** Definitions of key terminology.

Term			Definition
Page views			Number of people viewing a given web page
Click-through rate			Percentage of users who view and click on an advertisement (ad)
Conversions			An intended action on a website, such as downloads or purchases
Bounce rate			Percent of users leaving a website after viewing only 1 page
Conversion rate			Percentage of website sessions that lead to a desired activity
Session length			Amount of time a user spends on a website (in seconds)
User			An individual visitor to a website, including repeat visitors
Unique user			Nonduplicated visitors to a website
**Media channels**			
	Direct	Traffic that results from users directly entering the URL for a website	
	Organic search	Search results direct through a search engine	
	Paid search	Traffic that results from users clicking on paid search results	
	Social	Traffic driven through social media	

### Social Media and Email Targeting

The second phase of the quasi-experimental evaluation was dissemination via social media and email. Rather than using a broad dissemination pattern, the research team used a targeted dissemination approach on Facebook and Twitter.

For Facebook, the research team utilized CrowdTangle, Facebook’s research intelligence platform, to identify Facebook groups in New Jersey that topically focused on adolescent depression, mental health policy, or issues related to schools and mental health. CrowdTangle allows users to search by region and keyword. This approach identified 115 Facebook groups (43 were public and 72 were private). All group rules were followed, and research was conducted in accordance with IRB guidelines. Messages about the portal were posted in 73 Facebook groups (43 public and 30 private groups).

**Figure 4:**
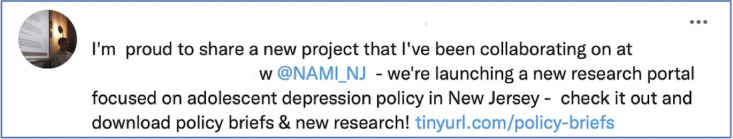
Sample of Twitter posting promoting the Project ASPEN portal.

To identify Twitter accounts, the research team leveraged the search feature enabled in the Twitter application programming interface. The team focused on searching for Twitter accounts that geoidentified as being from New Jersey and that used keywords represented in Table 2 as well as keywords specific to the legislation (eg, #A970). The resulting search aggregated 165,275 tweets from 16,437 users; the data were filtered for spam and other nonrelevant accounts using established keyword, location, and account ID filtering techniques. For instance, broadcast Twitter accounts such as @ParentalRights were eliminated from this analysis. This reduced the data set to 93,234 tweets from 11,203 users [40]. The top 100 Twitter accounts were selected; broadcast tweets such as the Tweet shown in Figure 4 were sent out by the project team as well as NAMI NJ.

**Table 2:** Sample keyword search terms.

Keyword group	Sample keywords
Depression	depression, adolescent depression, depression symptoms, depression screening tests, signs of depression
Mental health	youth mental health, high school mental health, school mental health
Policy	mental health policy, depression policy, depression policy resources, school mental health policy

In addition, direct messages were sent to the top 100 identified Twitter accounts promoting the portal (only 83 accounts allowed for direct messaging). The use of Twitter data builds on similar scholarship, for instance, following prior work examining Twitter and discussions pertaining to Medicaid [41].

Email accounts were identified based on a review of legislative activity, news media coverage, and social media content. News media and policy content relevant to the issue were tracked for the duration of 2021, and relevant individuals and organizations were identified on an ongoing basis. News media content was tracked by monitoring news stories on the topic of adolescent depression and suicide appearing in 12 local print and online news outlets, as well as television broadcast transcripts from New Jersey across the broad period of 2012-2020. Specific outlets tracked were Home News Tribune, Asbury Park Press, Courier-Post, Star-Ledger, Record, Trentonian, Press of Atlantic City, South Jersey Times, the Times, PR Newswire, NJ.Com, NJ Spotlight Press Releases, and NJ TV. A broader time range was used for the news media to aggregate enough articles (n=213).

Key policy makers were identified using Quorum (Quorum Analytics, Inc.). Quorum is a legislative tracking application that allows users to track policy at the state level. Using Quorum, the research team searched New Jersey legislative activity to identify state senators, assemblypersons, and staffers working on issues related to adolescent depression, following an approach established in similar studies tracking evidence use in policy making [42,43].

This cumulative analysis resulted in a list of 745 individuals (201 legislators and 544 policy advocates) who were involved in policy making and advocacy activities related to adolescent depression screening. Quorum was further used to retrieve email contact information. A web search was conducted for policy advocates. Of the initial list of 745 individuals, the research team was able to identify email addresses for 595 individuals (152 legislators and staffers and 443 policy advocates). Emails were sent using Constant Contact (Constant Contact, Inc.), an email tracking service, to track the open rate and click-throughs for the emails that were sent. We shared information with policy makers in their capacity as public officials and followed IRB guidelines for engagement.

### Targeted Dissemination Activity

The final stage of dissemination consisted of targeted dissemination activities. During this stage of the dissemination activity, the research team actively worked with community partners to spread the word about the knowledge portal and to share summaries of the research briefs in a PowerPoint (Microsoft Corporation) presentation format. Because of the ongoing COVID-19 pandemic, these presentations were run as webinars via Zoom (Zoom Video Communications, Inc.); all users were driven to the Project ASPEN website so that the project team could isolate traffic driven by these events. NAMI NJ worked with the research team to facilitate introductions to key groups and to facilitate presentations. Research presentations were made to the NAMI NJ Policy Advisory Board, NJ County Mental Health Board, NJ Behavioral Health Planning Council, NJ School Boards Association, Department of Education, and Department of Health.

### Statistical Analysis

Descriptive statistics were used to characterize user behavior and engagement with the website. A Poisson mean test was used to compare rates of use of the website across periods following prior related research. An unpaired 2-tailed *t* test was used to test difference for continuous variables. Variances are expressed as SDs.

## Results

### The Cumulative Campaign

**Figure 5:**
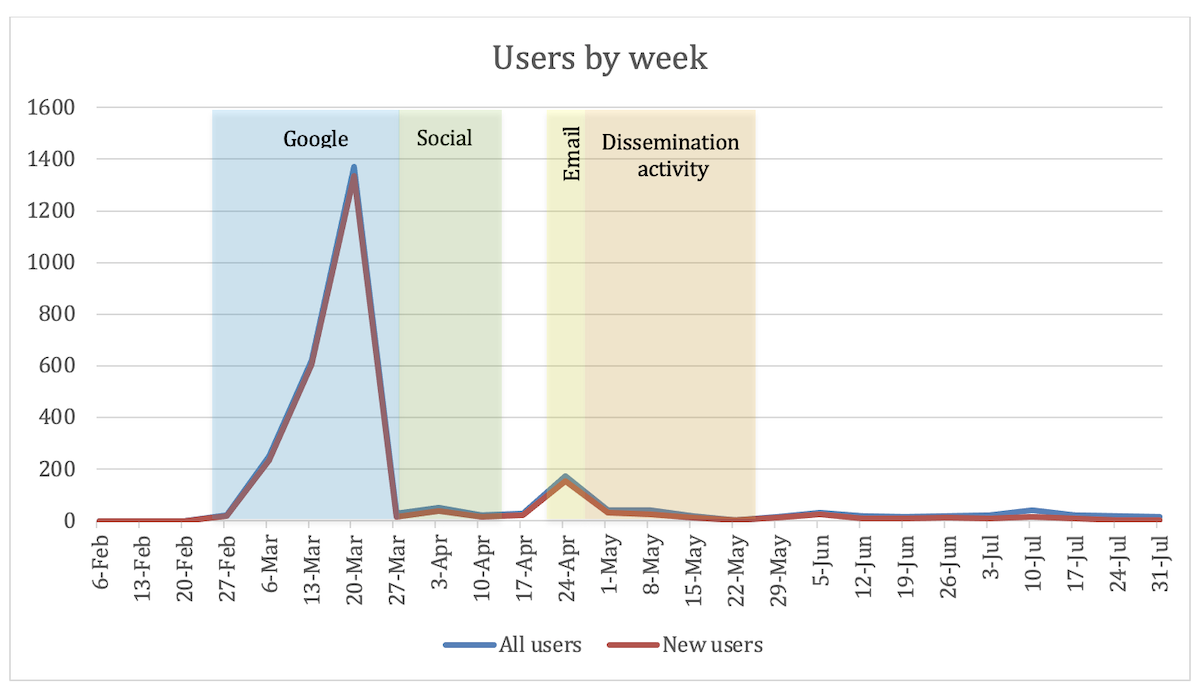
Summary of users by day.

The cumulative campaign ran for 161 days following the initial launch of the Project ASPEN knowledge portal. The knowledge portal was live for 21 days before promotional activity to establish a baseline with no promotional activity or outreach. The campaign resulted in 2837 unique user visits to the portal and 4713 page views. Daily counts of visits to the website from February 1, 2022, to July 31, 2022, are shown in Figure 5, with different promotional activities indicated by labels and color blocks.

**Figure 6:**
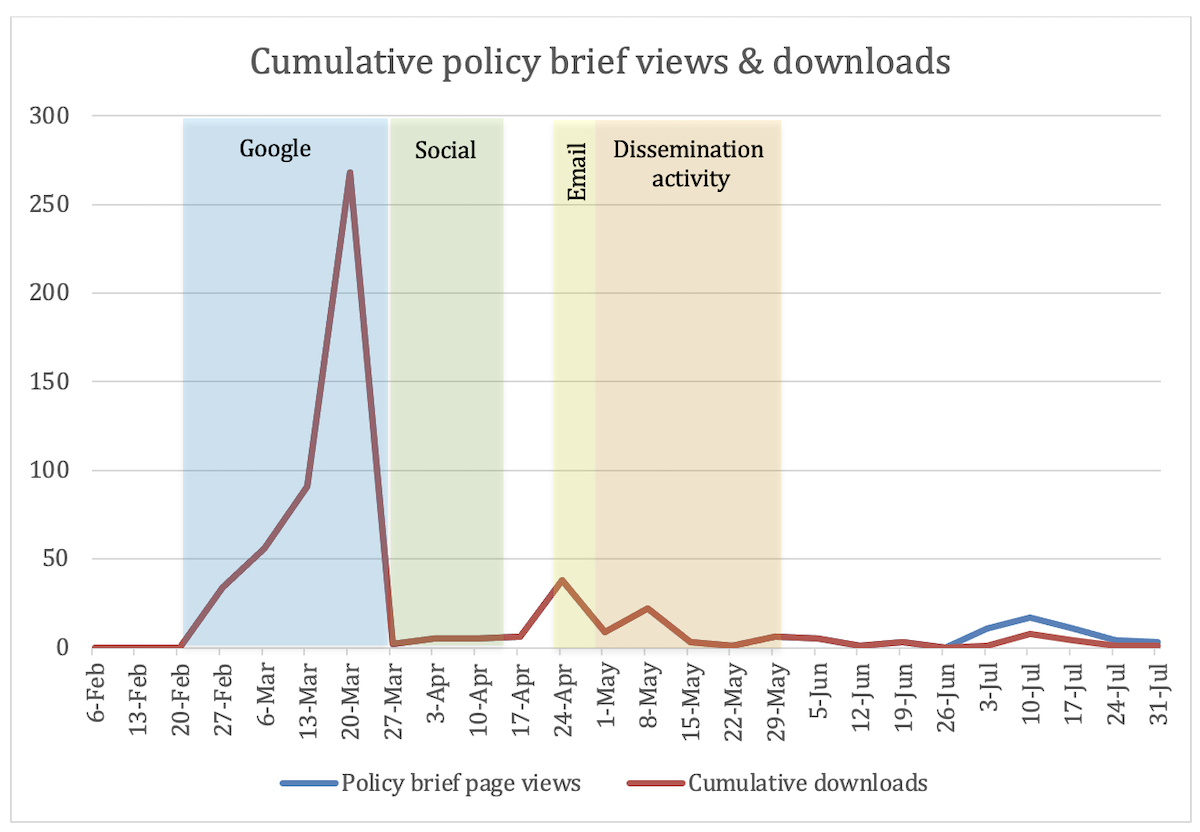
Summary of policy brief views and downloads by day.

Controlling for the number of days, the cumulative rate of visits increased significantly during the Google Ad campaign, with smaller bursts during the social media campaign, the email campaign, and the targeted dissemination activities period. The activity during each promotional period was substantially higher than the pre- and postcampaign periods when no promotional activity took place (Figure 6).

### Conversion Activity to Support Knowledge Brokering

The core objective of utilizing a website platform to support knowledge brokerage activity was to drive user engagement with policy briefs that were supplied via the portal. The website was designed to allow users to engage with policy briefs in 2 different ways: users could view the policy briefs directly on the web page or download a PDF version of the policy brief from the same web page.

There were no policy brief page views or downloads prior to the start of the promotional activity. Between the start of the Google Ads campaign on February 27, 2022, and the end of the intentional and targeted knowledge brokering activity on May 31, 2022, there were 588 page views within the policy brief section of the knowledge portal, and 59 downloads of PDF versions of the policy briefs. Overall, this translates to a 19.82% (449/2265) conversion rate for users to policy brief page views, and a 1.50% (34/2265) conversion rate for users to policy brief downloads.

### Comparison of Conversion Activity by Promotional Channel

Table 3 summarizes the differences in conversion rates by promotional activity type.

**Table 3:** Summary of campaign by activity type.

Activity type	Users, n	Policy brief views, n	Policy brief downloads, n	Policy brief view conversion, n/N (%)	Download conversion, n/N (%)
Google Ads	2265	449	34	19.8 (449/2265)	1.5 (34/2265)
Email	175	38	7	21.7 (38/175)	4.0 (7/175)
Social media	131	18	2	14.9 (18/121)	1.5 (2/131)
Targeted dissemination activities	204	50	14	24.5 (50/204)	6.9 (14/204)
Postlaunch	62	16	2	25.8 (16/62)	3.2 (2/62)

The rate (views/day) of policy brief page view conversions was significantly higher for Google Ads compared with email (16.0 vs 5.4; *P*<.001), social media (16.0 vs 0.6; *P*<.001), knowledge brokering activities (16.0 vs 0.8; *P*<.001), and the postlaunch period (16.0 vs 1.1; *P*=.009). The number of policy brief page view conversions for the email campaign was significantly higher compared with social media (5.4 vs 0.6; *P*<.001), knowledge brokering activities (5.4 vs 0.8; *P*<.001), and the postlaunch period (5.4 vs 1.1; *P*=.001). Similarly, the download conversion rate for Google Ads was significantly higher compared with social media (1.2 vs 0.1; *P*<.001), knowledge brokering activity (1.2 vs 0.2; *P*<.001), and the postlaunch period (1.2 vs 0.1; *P*<.001). There was no significant difference in the download conversion rate between the Google Ads campaign and the email campaign (1.2 vs 1.0; *P*=.54). The download conversion rate for the email campaign was significantly higher than the rate for social media (1.0 vs 0.1; *P*<.001), knowledge brokering activity (1.0 vs 0.2; *P*<.001), and the postlaunch period (1.0 vs 0.1; *P*<.001).

### Google Ads Results

Looking specifically at the results from the Google Ads campaign, there are some differences in user behavior in this period that are important to note. During the Google Ads campaign, 98% of traffic to the portal was driven by paid search results (2219 user visits), with the remaining 2% coming from organic search. The average daily user count was higher during the campaign than during other periods (80.9 vs 4.9; *P*<.001). The number of pages viewed, however, was lower per session than during other periods (mean 1.7, SD 0.3 vs mean 1.8, SD 0.5; *P*<.001). The session length during the Google Ads campaign was significantly lower than that during other periods (mean 33, SD 15 vs mean 53, SD 32; *P*<.001).

In summary, the Google Ads campaign drove a significant number of visits to the portal, and those visits resulted in a higher conversion rate in terms of both policy brief page views and policy brief downloads. The number of pages viewed by each user was lower and the amount of time spent on the site was also lower.

### Keyword Behavior

The average cost per click for the campaign was US $2.09. A summary of clicks and the associated cost per click are shown in Table 4 for the top 10 search keywords. The analytics from the Google Ads campaign allows campaign managers to view differences based on specific terms. The results show that depression- and symptom-related terms were the most notable drivers of traffic to the website. Notably, the term “depression screening test” had the highest click-through rate, indicating that users were particularly responsive to that search term. The broad term of “depression” drove the most traffic in terms of clicks.

**Table 4:** Summary of keyword ad results.

Search keyword	Clicks, n	Cost per click (US $)	Click-through rate, n/N (%)	Bounce rate, %
depression	791	2.11	3.40 (791/23,264)	83.6
depression symptoms	469	2.12	5.77 (469/8128)	84.2
depression screening test	371	2.07	11.69 (371/3174)	88.6
teen depression	210	2.13	8.23 (210/2552)	85.2
bipolar	189	1.92	4.21 (189/4489)	92.3
signs of depression	97	2.14	5.08 (97/1909)	80.3
depression and anxiety	85	2.08	4.15 (85/2048)	88.9
policy	54	1.98	2.68 (54/2015)	79.8
adolescent depression	42	2.27	5.62 (42/747)	83.5
major depression	37	2.13	5.63 (37/657)	88.1

Bounce rate gives an indication of the percent of users that leave the website after viewing the landing page. For instance, the search term “policy” had the lowest bounce rate (79.8%), indicating that users who clicked on that keyword were more likely to stay on the website compared with users clicking on other search terms. Overall, the results from the Google Ads campaign had a higher bounce rate than for the other promotional periods (86.0% vs 79.3%; *P*=.008).

### Social Media Results

The social media campaign targeted 73 Facebook groups and 100 targeted Twitter accounts, as well as posted broadly targeted messages on Twitter and Facebook. The social media campaign drove 131 users to the portal, resulting in 18 policy brief page views and 2 policy brief downloads. Traffic was relatively split, with 72 users driven by Facebook activity and 59 users driven by Twitter. The subgroups were not large enough to warrant analysis based on specific social media channel.

There was no significant difference in the daily user count during the social media campaign compared with other periods, excluding the Google Ads campaign (mean 4.8, SD 0.8 vs mean 4.7, SD 3.1; *P*=.86). The number of pages viewed similarly was not significant (mean 10.2, SD 6.5 vs mean 8.9, SD 2.3; *P*=.37).

### Email Results

During the email campaign, 595 emails were sent out to 152 legislators and staffers, and 443 policy advocates. The email campaign had an open rate of 50.9% (303 emails: 71 legislators and staffers and 232 policy advocates), which is notably high and likely due to the targeted nature of the emails. The email campaign resulted in 38 policy brief page views and 7 policy brief downloads.

During the email campaign there was a significantly higher number of daily users compared with other periods, excluding the Google Ads campaign (mean 25.0, SD 4.2 vs mean 3.6, SD 0.7; *P*<.001). There was no significant difference in terms of the number of page views (mean 2.2, SD 1.2 vs mean 1.5, SD 1.1; *P*=.05).

### Targeted Dissemination Activities Results

The targeted dissemination activities focused on research presentations to key stakeholders to drive awareness of the knowledge portal and to encourage use of the information on the portal. This knowledge brokering campaign drove 204 users to the portal, resulting in 50 policy brief page views and 14 policy brief downloads over a 63-day period. There was a significantly lower number of daily users compared with other periods, excluding the Google Ads campaign (mean 3.2, SD 2.1 vs mean 6.6, SD 4.0; *P*<.001). There was also a significant difference in terms of the number of page views (mean 2.1, SD 0.6 vs mean 1.3, SD 1.1; *P*<.001).

### Role of Geography and Technology

Based on overall traffic, 93% of all traffic came from locations in the United States; 72% came from locations broadly identified as New York City metro (primarily New Jersey), 68% of users came from mobile devices and 32% of users came from desktop computers. Looking specifically at traffic driven by the Google Ads campaign, 78% of users came from mobile devices and 22% of users came from desktop computers.

## Discussion

### Overview

Results from this research compare the use of various dissemination strategies for creating engagement and brokering research evidence in the process of policy implementation.

### Principal Findings

This is one of the first studies to explicitly design, implement, and test the efficacy of a knowledge portal as a means of supporting knowledge brokerage, and to compare a variety of approaches for facilitating use of the knowledge portal. Our findings show that Google Ads can be utilized to drive a substantial increase in the number of visitors to a website. While Google Ads was effective in driving both traffic and conversions through policy web page views and policy brief downloads, the amount of time users spent on the site and the number of pages viewed on average was lower than traffic driven by other sources.

Moreover, driving traffic with Google Ads is a relatively inefficient way to drive traffic; the average cost per click of US $2.09 means that significant expense is needed to drive large volumes of users. As noted previously [44], a common comparison method for Google Ad campaigns is to measure effectiveness based on the cost per conversion for the desired activity. For Project ASPEN, the core activity was policy brief views and downloads, and the cost per conversion was US $11.1 per policy page view and US $147.1 per policy download. Comparatively, a recent work in other Google Ads campaigns related to health suggested costs per conversion ranging from US $6.70 to US $55 [31]. As others have noted, while the cost per conversion ultimately compares favorably to broader advertising campaigns designed to generate revenue, this type of model is not sustainable for driving use of research evidence.

In part, the ineffectiveness of Google Ads is likely because paid search targets a broad audience based on keywords. Therefore, the users who are clicking on the selected keywords are likely searching for broader information about depression and anxiety. Prior work echoes this, showing that policy makers are likely to turn to trusted peers and other known sources of knowledge when seeking policy-relevant information [45].

Other targeted approaches proved effective in driving conversions and generating policy views and downloads without significant expense. Specifically, the use of targeted emails was effective in terms of the rate of policy views and the rate of downloads. The findings for targeted emails are somewhat inflated because the emails were sent over a concentrated period (1 week) resulting in an artificially high rate; in terms of raw volume of policy views and downloads, the process of targeted dissemination activities was more effective but more time-consuming. Open rates for the targeted dissemination via email exceed rates from a prior study that examined policy making and the adolescent mental health context (open rate of 50.9%, 303/595, in this study vs 22.8% in the referenced study, resulting in a 22.3% policy brief view rate) [46]. A similar work found an open rate of 20.3%-40.5% based on a series of trials related to COVID-19 policies [43].

Our higher open rate is likely due to the targeted nature of the campaign. The targeted emails were sent with a specific message encouraging legislators and policy advocates to access information that was tailored to their needs and were also sent using university branding. This echoes prior work suggesting email can be effective for engaging policy makers [47]; that work also points to a need for better targeting strategies, which this work validates.

The targeted dissemination activities that took place were customized for each audience; the small group settings and the time taken during the sessions to highlight policy-relevant information likely translated directly to policy web page views and policy brief downloads. Indeed, multiple participants in the targeted dissemination sessions remarked that they would share the knowledge portal with their colleagues.

Social media was not effective for a multitude of reasons. The social media environment is cluttered with a multitude of advertisements. As a result, the messages that were sent during the campaign to support the knowledge portal were likely received with myriad other communications.

In summary, Google Ads, targeted email communication, and targeted dissemination sessions were successful in driving conversion as policy web page views and policy brief downloads. More broadly, the Project ASPEN knowledge portal served as a useful site for driving knowledge brokerage in the form of web page views of the policy briefs. As noted, it was harder to drive actual downloads of the PDF, but extant research supports the notion that the success rate of a desired online activity will go down as the number of steps required to accomplish the activity go up. Based on the degree of activity on the website, the effectiveness in reaching geographically relevant users, and engagement with policy brief views and downloads, we are optimistic about the utility of this approach as a means of driving awareness of policy-relevant research.

### Limitations

The findings from this study are limited to this specific application and case study involving pending implementation of the school-based adolescent depression screening program in the state of New Jersey. The political context of each state is likely to vary, as is the policy implementation process. Thus, what works in one setting may not work in another. Besides, we did not manipulate message design; there is evidence that intentional design such as the use of emotion in messaging can improve response rates to policy communication [48]. Nevertheless, the concept of online knowledge portals as brokers has the potential to enable brokerage activity. There are limitations in what can be tracked with the platforms utilized, and as a result some of the descriptions of user activities are somewhat limited. It is hard to ascertain who specifically downloaded policy briefs or policy outcomes. Rather, by focusing on dissemination in key phases, this study attempted to narrow who was exposed to the knowledge portal, but nevertheless this remains a notable limitation. Moreover, this study was unable to determine what happened with knowledge retrieved from the portal beyond user engagement and downloads.

### Conclusions

Our study is the first to empirically test the concept of a website as a resource that supports activity related to knowledge brokerage in the context of health policy implementation. The findings highlight many of the challenges associated with such research in practice, but also point toward key pathways for success in implementation, including the use of targeted email lists and keywords paired with website design for successful implementation. As researchers and policy makers continue to turn to online sources to both locate and disseminate information, it is increasingly critical to understand how websites can function as platforms for knowledge brokering activity.

## Abbreviations

ASPEN: Active Surveillance of Policy Ecosystems and Networks
IRB: institutional review board

## Data Availability

The data sets generated during or analyzed during this study are available from the corresponding author on reasonable request.

## References

[1] Ward V, House A, Hamer S (2009). Knowledge Brokering: The missing link in the evidence to action chain?. Evid Policy.

[2] Meyer M (2010). The Rise of the Knowledge Broker. Science Communication.

[3] Innvaer Simon, Vist G, Trommald M, Oxman A (2002). Health policy-makers' perceptions of their use of evidence: a systematic review. J Health Serv Res Policy.

[4] Jordan ME, Lanham HJ, Crabtree BF, Nutting PA, Miller WL, Stange KC, McDaniel RR (2009). The role of conversation in health care interventions: enabling sensemaking and learning. Implement Sci.

[5] Brown A, Barnes C, Byaruhanga J, McLaughlin M, Hodder RK, Booth D, Nathan N, Sutherland R, Wolfenden L (2020). Effectiveness of Technology-Enabled Knowledge Translation Strategies in Improving the Use of Research in Public Health: Systematic Review. J Med Internet Res.

[6] Yanovitzky I, Weber M (2019). Analysing use of evidence in public policymaking processes: a theory-grounded content analysis methodology. Evid Policy.

[7] Ward VL, House AO, Hamer S (2009). Knowledge brokering: exploring the process of transferring knowledge into action. BMC Health Serv Res.

[8] Lavis J, Robertson D, Woodside J, McLeod C, Abelson J, Knowledge Transfer Study Group (2003). How can research organizations more effectively transfer research knowledge to decision makers?. Milbank Q.

[9] Lawlor J, Hammond J, Lagoze C, Huynh M, Moss P, Weber MS, Yanovitzky I (2021). Platformed Knowledge Brokerage in Education: Power and Possibilities. Knowledge Brokers, Networks, the Policymaking Process.

[10] Dobbins M, Hanna SE, Ciliska D, Manske S, Cameron R, Mercer SL, O'Mara L, DeCorby K, Robeson P (2009). A randomized controlled trial evaluating the impact of knowledge translation and exchange strategies. Implement Sci.

[11] Jeong D, Cheng M, St-Jean M, Jalali A (2019). Evaluation of eMentalHealth.ca, a Canadian Mental Health Website Portal: Mixed Methods Assessment. JMIR Ment Health.

[12] Sharratt M, Usoro A (2003). Understanding knowledge?sharing in online communities of practice. Electronic Journal of Knowledge Management.

[13] Gough D, Maidment C, Sharples J (2021). Enabling knowledge brokerage intermediaries to be evidence-informed. Evid Policy.

[14] Waardenburg L, Huysman M, Sergeeva AV (2022). In the Land of the Blind, the One-Eyed Man Is King: Knowledge Brokerage in the Age of Learning Algorithms. Organization Science.

[15] Loncarevic N, Andersen PT, Leppin A, Bertram M (2021). Policymakers' Research Capacities, Engagement, and Use of Research in Public Health Policymaking. Int J Environ Res Public Health.

[16] Ashcraft LE, Quinn DA, Brownson RC (2020). Strategies for effective dissemination of research to United States policymakers: a systematic review. Implement Sci.

[17] Supovitz J, Daly AJ, Del Fresno M (2018). The Common Core debate on Twitter and the rise of the activist public. J Educ Change.

[18] Wang Y, Fikis DJ (2017). Common Core State Standards on Twitter: Public Sentiment and Opinion Leaders. Educational Policy.

[19] Hammill A, Harvey B, Echeverria D (2013). Knowledge for action: an analysis of the use of online climate knowledge brokering platforms. Knowledge Management for Development Journal.

[20] Pang P, Chang S, Clavisi O, editors (2020). Intention of Use of the Patient-centric Research Engagement Portal (PREP). PACIS 2020 Proceedings.

[21] Gluckman PD, Bardsley A, Kaiser M (2021). Brokerage at the science–policy interface: from conceptual framework to practical guidance. Humanit Soc Sci Commun.

[22] Pang PC, Chang S, Verspoor K, Clavisi O (2018). The Use of Web-Based Technologies in Health Research Participation: Qualitative Study of Consumer and Researcher Experiences. J Med Internet Res.

[23] Burchill C, Roos LL, Fergusson P, Jebamani L, Turner K, Dueck S (2000). Organizing the present, looking to the future: an online knowledge repository to facilitate collaboration. J Med Internet Res.

[24] Loebbecke C, Crowston K (2012). Components, functionalities,deployment challenges. Proceedings of the Thirty Third International Conference on Information Systems.

[25] Chau M, Huang Z, Qin J, Zhou Y, Chen H (2006). Building a scientific knowledge web portal: The NanoPort experience. Decision Support Systems.

[26] Plaza B (2011). Google Analytics for measuring website performance. Tourism Management.

[27] Spoelman WA, Bonten TN, de Waal MWM, Drenthen T, Smeele IJM, Nielen MMJ, Chavannes NH (2016). Effect of an evidence-based website on healthcare usage: an interrupted time-series study. BMJ Open.

[28] Tieman J, Bradley SL (2013). Systematic review of the types of methods and approaches used to assess the effectiveness of healthcare information websites. Aust. J. Prim. Health.

[29] Barbosa B, Oliveira Z, Teixeira S, Gomes V, Saura JR (2021). Understanding Google Ads Metrics for SME. Advanced Digital Marketing Strategies in a Data-Driven Era.

[30] Jessup DL, Glover Iv McKinley, Daye D, Banzi L, Jones P, Choy G, Shepard JO, Flores EJ (2018). Implementation of Digital Awareness Strategies to Engage Patients and Providers in a Lung Cancer Screening Program: Retrospective Study. J Med Internet Res.

[31] Murphy AL, Peltekian S, Gardner DM (2018). Website Analytics of a Google Ads Campaign for a Men's Mental Health Website: Comparative Analysis. JMIR Ment Health.

[32] Rolls K, Hansen M, Jackson D, Elliott D (2016). How Health Care Professionals Use Social Media to Create Virtual Communities: An Integrative Review. J Med Internet Res.

[33] Krall J (2013). Using social metrics to evaluate the impact of online healthcare communications. Journal of Communication in Healthcare.

[34] Young HM, Bell JF, Tonkikh O, Kilaberia TR, Whitney RL, Mongoven JM, Link BM, Kelly K (2022). Implementation of a Statewide Web-Based Caregiver Resource Information System (CareNav): Mixed Methods Study. JMIR Form Res.

[35] Johnson D, Dupuis G, Piche J, Clayborne Z, Colman I (2018). Adult mental health outcomes of adolescent depression: A systematic review. Depress Anxiety.

[36] Siu AL (2016). Screening for Depression in Children and Adolescents: U.S. Preventive Services Task Force Recommendation Statement. Ann Intern Med.

[37] Sekhar D, Ba D, Liu G, Kraschnewski J (2019). Major Depressive Disorder Screening Remains Low Even Among Privately Insured Adolescents. J Pediatr.

[38] Riehm K, Brignone E, Stuart E, Gallo J, Mojtabai R (2022). Diagnoses and Treatment After Depression Screening in Primary Care Among Youth. Am J Prev Med.

[39] Kazdin AE, Blase SL (2011). Rebooting Psychotherapy Research and Practice to Reduce the Burden of Mental Illness. Perspect Psychol Sci.

[40] Chen K, Duan Z, Yang S (2023). Twitter as research data. Politics Life Sci.

[41] Zhu JM, Sarker A, Gollust S, Merchant R, Grande D (2020). Characteristics of Twitter Use by State Medicaid Programs in the United States: Machine Learning Approach. J Med Internet Res.

[42] Scott JT, Prendergast S, Demeusy E, McGuire K, Crowley M (2022). Trends and Opportunities for Bridging Prevention Science and US Federal Policy. Prev Sci.

[43] Scott T, Pugel J, Fernandes M, Cruz K, Long E, Giray C (2022). Cutting through the noise during crisis by enhancing the relevance of research to policymakers. Evid Policy.

[44] Gordon JS, Akers L, Severson HH, Danaher BG, Boles SM (2006). Successful participant recruitment strategies for an online smokeless tobacco cessation program. Nicotine Tob Res.

[45] Bogenschneider K, Day E, Bogenschneider BN (2021). A window into youth and family policy: State policymaker views on polarization and research utilization. Am Psychol.

[46] Purtle J, Nelson KL, Gebrekristos L, Lê-Scherban Félice, Gollust SE (2022). Partisan differences in the effects of economic evidence and local data on legislator engagement with dissemination materials about behavioral health: a dissemination trial. Implement Sci.

[47] Pugel J, Long E, Fernandes M, Cruz K, Giray C, Crowley D, Scott Taylor (2022). Who is listening? Profiles of policymaker engagement with scientific communication. Policy Internet.

[48] Long EC, Pugel J, Scott JT, Charlot N, Giray C, Fernandes MA, Crowley DM (2021). Rapid-Cycle Experimentation With State and Federal Policymakers for Optimizing the Reach of Racial Equity Research. Am J Public Health.

